# Positivity to Cocaine and/or Benzoylecgonine in Confirmation Analyses for On-Road Tests in Spain

**DOI:** 10.3390/ijerph18105371

**Published:** 2021-05-18

**Authors:** Francisco Herrera-Gómez, Eduardo Gutiérrez-Abejón, Mercedes García-Mingo, F. Javier Álvarez

**Affiliations:** 1 Institute for Alcohol and Drug Studies, Pharmacology and Therapeutics, Faculty of Medicine, University of Valladolid, 47005 Valladolid, Spain; fherrerag@saludcastillayleon.es (F.H.-G.); mercedes.garcia.mingo@uva.es (M.G.-M.); alvarez@med.uva.es (F.J.Á.); 2 Pharmacological Big Data Laboratory, Pharmacology and Therapeutics, Faculty of Medicine, University of Valladolid, 47005 Valladolid, Spain; 3 Department of Nephrology, Hospital Virgen de la Concha, 49022 Zamora, Spain; 4 Technical Direction of Pharmaceutical Assistance, Gerencia Regional de Salud de Castilla y León, 47007 Valladolid, Spain; 5 CEIm, Hospital Clínico Universitario de Valladolid, 47003 Valladolid, Spain

**Keywords:** automobile driving, cocaine, driving under the influence, epidemiology, oral fluid, psychotropic drugs, saliva, street drug testing, substance abuse detection

## Abstract

We are using real-life data in order to determine the prevalence of driving with the presence of cocaine and/or benzoylecgonine (BZE), their concentrations, and their use in combination with other drugs. This study assessed data on Spanish drivers with confirmed drug-positive results recorded by the Spanish National Traffic Agency from 2011–2016. Frequencies of positivity for cocaine and/or BZE and concentration of such substances were obtained. Comparisons and univariate and multivariate regression analyses were performed. Drivers who tested positive for cocaine and/or BZE accounted for 48.59% of the total positive results for drugs. In positive cases for both cocaine and BZE, other substances were detected in 81.74%: delta-9-tetrahydrocannabinol (THC) (68.19%), opioids (20.78%) and amphetamine-like substances (16.76%). In the multivariate logistic regression analysis, the frequency of cocaine and/or BZE positive cases decreased with age (OR:0.97) and were less likely among women (OR:0.63). Concentrations (ng/mL) of cocaine (249.30) and BZE (137.90) were higher when both substances were detected together than when detected alone. Positivity to cocaine represented an important proportion among Spanish drivers who tested positive for drugs, and polysubstance use was especially observed in more than 8 out of 10 positive cases for cocaine and/or BZE.

## 1. Introduction

Cocaine is a well-known driving-impairing substance [1]. The use of cocaine is associated with a medium increased risk (relative risk of 2 to 10) of being seriously injured or killed in a traffic accident, according to findings from the Driving Under the Influence of Drugs, alcohol and medicines (DRUID) project [2]. Such a risk is similar to that of driving with a blood alcohol concentration in the range of 0.5 g/L to 0.8 g/L. The recorded estimation for accident involvement after consuming cocaine was 2.96 (95% CI 1.18–7.38); for involvement in fatal accidents it was 1.66 (95% CI 0.91–3.02); and for injuries in accidents it was 1.44 (95% CI 0.93–2.23) [3]. Furthermore, multiple drug use involving cocaine is associated with a very increased risk [2,3,4], thus, constituting a great concern, especially if alcohol consumption is detected.

Worldwide, driving under the influence of alcohol, illicit drugs and certain medicines is not allowed [1], and regulations follow three well-defined legal approaches: (i) zero tolerance, that is, it is unlawful to drive with any amount of driving-impairing substances in the body; (ii) impairment, that is, it is illegal to drive when one is impaired due to such drugs, or ‘under the influence’; and (iii) per se, that is, a maximum set concentration above which it is unlawful to drive is defined [1].

In Spain, we have a dual legal approach: zero-tolerance and impairment laws apply [5]. According to our zero-tolerance system, the driver is punished when any amount of drug is detected (except prescribed medicines used according to medical indications); impairment of driving abilities is not required. In the absence of impairment, only administrative sanctions are imposed on the infringing driver (a fine of EU1000 along with the loss of 6 driving license points). On the other hand, when impairment signs due to psychoactive drugs are observed, the driver is punished as a criminal offender (imprisonment for 3–6 months, a fine, or community service of 31–90 days with, in all cases, driving disqualification for 1–4 years).

Oral fluid (OF) is a good alternative biological matrix to test drug use at roadside or in drivers involved in car crashes, providing immediate evidence of driving with the presence of this drug [6]. In the past years, OF drug testing has become a reality in many developed countries. Nevertheless, sensitivity and specificity are still a matter of concern [6,7,8,9,10]. Furthermore, accurate quantification of drugs detected on the roadside requires a two-step drug detection procedure: on-road screening testing is followed by analysis of confirmation and quantification of the detected substances in toxicology laboratories when a roadside drug test is positive.

Cocaine is largely metabolized into the body, with benzoylecgonine (BZE) being the major inactive metabolite [11,12]. Current OF roadside drug testing and laboratory confirmation analyses detect cocaine and their main inactive metabolite BZE. At least in Spain, sanctions are imposed when cocaine (with and without BZE) is confirmed at the laboratory but not when only BZE (without cocaine) is detected, because this last is an inactive metabolite.

This study used real-world data (i.e., results of OF drug analysis from toxicological laboratories analyses confirming positivity to drugs on the roadside in Spain) to determine the prevalence of driving with the presence of cocaine and/or BZE, their use in combination with other drugs, concentrations of cocaine and/or BZE, and to analyze the association of these factors with age and gender.

## 2. Materials and Methods

As previously performed [5,13,14], this study assessed Spanish National administrative data on laboratory-confirmed drug-positive results obtained from the Spanish National Traffic Agency records corresponding to licensed drivers who underwent drug confirmation analyses between 1 January 2011, and 31 December 2016. Hypothesis, analysis and reporting follow the REporting of studies Conducted using Observational Routinely collected health Data (RECORD) guidelines [15]. This study was approved by the Ethics Review Board, CEIm Área de Salud Valladolid Este, on 28 September 2017 (Reference number PI 17–814).

In Spain, mandatory roadside alcohol and drug testing (screening) are carried out by the Spanish Traffic Police using breath for alcohol (Dräger Alcotest^®^ 6810 device; Drägerwerk AG & Co. KGaA, Lübeck, Germany) and oral fluid for drugs (Dräger DrugTest^®^ 5000; Drägerwerk AG & Co. KGaA, Lübeck, Germany, DrugWipe^®^; Securetec Detektions-Systeme AG, Neubiberg, Germany, or Alere™ DDS^®^2 Mobile Test System; Alere Toxicology Services Inc., Los Angeles, CA, USA). All positive results for any substance other than alcohol need to be confirmed and quantified, so a second oral fluid sample of approximately 1 mL is obtained and sent to accredited toxicology laboratories for confirmation analysis and quantification of detected substances using chromatographic techniques [5,13,14]. Supplementary Table S1 shows the cut-offs for roadside drug tests for cannabis, cocaine, amphetamine, methamphetamine and opioids that have been used between 2011 and 2016. Confirmed positive drug tests are then recorded at the Spanish National Traffic Agency (Dirección General de Tráfico).

A total of 179,645 roadside drug tests were carried out by the Spanish traffic police between 2011 and 2016 (Supplementary Table S2), 65,244 of which were positive [5,13,14]. Supplementary Table S2 shows information on gender distribution in the Spanish Driver population between 2011 and 2016.

The following groups of licit/illicit drugs and some of their metabolites were assessed in confirmation and quantification analyses according to DRUID project criteria [2,5,13,14]: (1) amphetaminelike substances (amphetamine, 3,4-methylenedioxymethamphetamine (MDMA), 3,4-methylenedioxyamphetamine (MDA), 3,4-methylenedioxy-N-ethylamphetamine (MDEA), methamphetamine); (2) cocaine and benzoylecgonine; (3) delta-9-tetrahydrocannabinol (THC); (4) opioids (6-acetylmorphine, morphine, codeine, methadone, tramadol); (5) benzodiazepines (hypnotics: flunitrazepam, 7-aminoflunitrazepam; anxiolytics: alprazolam, clonazepam, 7-aminoclonazepam, diazepam, lorazepam, nordiazepam, oxazepam); and (6) z-drugs (zolpidem, zopiclone).

Any positive result for a given substance was considered a positive case when the concentration for such substance was higher than the lowest limit of quantification using liquid chromatography or gas chromatography with mass spectrometry. No information on alcohol was accessible. The lowest limit of quantification for cocaine and BZE assessed was 0.5 ng/mL in OF.

The anonymized data set provided by the Spanish National Traffic Agency contained the following information for each positive case: (1) date of the drug test, (2) age and gender, and (3) concentration of detected substances (in all cases, in ng/mL).

The accessed dataset did not include information regarding results on breath-alcohol tests. Because the dataset is being used for administrative purposes, for many drivers no information was recorded on age and gender, and this information was only available for the year 2016 (Supplementary Table S2). Furthermore, for negative roadside drug tests, no information was recorded, according to the data protection regulation in Spain.

The prevalence of cocaine and BZE use in the study population (frequencies expressed as percentage) was obtained from confirmed positive tests, according to the age and gender of participants (Supplementary Table S2). Cocaine and BZE concentrations are presented as mean with their corresponding standard deviation, and medians with their quartiles (Q) 1 and 3. Decile distribution of cocaine and BZE concentration were also calculated. Differences by gender and age were determined using the chi-square test (χ^2^) for categorical variables and Kruskal–Wallis H test for continuous variables.

Multivariate regression analyses were performed to evaluate the relationships of positive cases for cocaine and/or BZE with age (as a continuous variable), gender, and the interactions between age and gender, for which odds ratios (ORs) with their corresponding 95% confidence interval (95% CI) are presented. The significance level was set at *p* ≤ 0.05. The Statistical Package for the Social Sciences (SPSS version 24.0.; SPSS Inc, Chicago, IL, USA) was used for the statistical analysis.

## 3. Results

Drivers positive for cocaine and/or BZE accounted for 48.59% of the tested drivers (31,707 out of 65,244). Cocaine- and BZE-positive cases were a common finding (39.50%, *n* = 25,773), while positive cases for cocaine without BZE were less frequently observed (8.33%, *n* = 5436), and positive cases for BZE without cocaine were uncommon (0.76%, *n* = 498) (Table 1).

In the majority of drivers who tested positive for cocaine and/or BZE, other substances were also detected: 81.74% of drivers positive for both cocaine and BZE were positive for other substances, particularly THC (68.19%), and less frequently opioids (20.78%) and amphetaminelike substances (16.76%). The same proportions of combined use were also observed among those positive for cocaine—without BZE—and those positive for BZE—without cocaine (Table 1).

**Figure 1:**
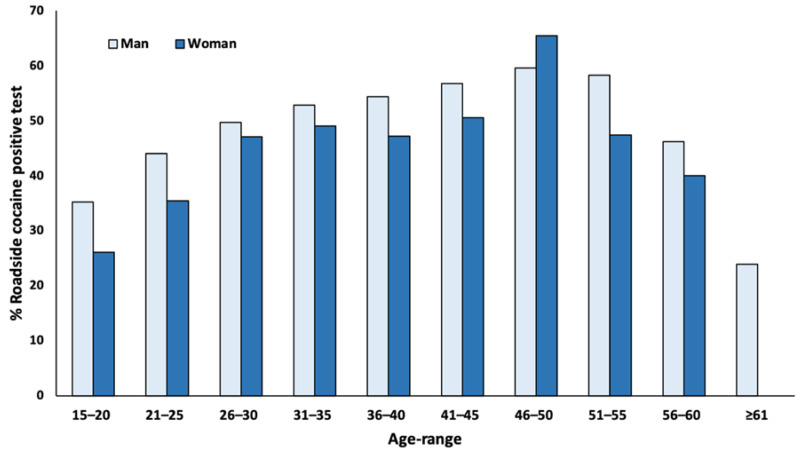
Distribution by age and gender of the confirmed positive oral fluid tests for cocaine and/or benzoylecgonine (years 2011–2016).

Drivers who tested positive for cocaine and/or BZE were commonly males than females (48.36% (16,780 out of 34,691) versus 43.79% (586 out of 1338), X^2^ = 10.79, *p* < 0.0001). In addition, most cases of positivity for cocaine and/or BZE occurred among drivers in the age groups of 21–25 (23.39%), 26–30 (25.26%) and 31–35 (19.27%). Figure 1 shows the occurrence of these positive cases by age and gender: Except for those younger or older, nearly half were positive.

In the multivariate logistic regression analysis, the frequency of positive cases for cocaine and/or BZE decreased with age (OR, 0.97; 95% CI, 0.95–0.98; *p* < 0.0001), and was less common among women (OR, 0.63; 95% CI, 0.40–0.99; *p* = 0.046), but the interaction between age and gender showed any effect (OR, 1.01; 95% CI, 0.99–1.02; *p* = 0.251).

Concentrations (median, ng/mL) of cocaine (249.30) and BZE (137.90) were higher when both substances were detected together than when cocaine—without BZE—(11.00, *p* < 0.0001) and BZE—without cocaine—(9.90, *p* < 0.0001) were detected alone (Table 2). Supplementary Table S3 shows the decile concentration distribution of cocaine- and BZE-positive cases confirmed at toxicology laboratories. A boxplot presents distribution of cocaine and BZE concentrations by 5-year age in Supplementary Figures S1 and S2; an important dispersion in cocaine and BZE concentrations was observed.

## 4. Discussion

Drivers who tested positive for cocaine and/or BZE represented an important proportion among confirmed roadside drug tests performed from 2011 to 2016. Indeed, in nearly half of the cases cocaine and/or BZE was present. Cocaine was just the second most frequent detected drug, after cannabis (79.5%) [13].

Our findings depict also how frequent polydrug use is in our country: In over 8 out of 10 positives cases for cocaine and/or BZE, other substances apart from alcohol, particularly cannabis, are confirmed. The increased accident risk when driving with the presence of other drugs is well known. This finding is consistent with polysubstance use observed when assessing confirmed tests for opioids and cannabis in the same period in Spain [13,14] and worldwide [2,4,16,17,18].

Nevertheless, the number of tests decreased with age, and they were uncommon among women. This age and gender relationship is well known regarding substance use and driving with the presence of drugs [5,13].

The percentage of positive results for cocaine and/or benzoylecgonine is similar to other national studies [10,19,20,21] and higher than in other DRUID project member countries [2].

Cocaine is rapidly metabolized. Therefore, it is not surprising that in most cases (81%) was confirmed in OF cocaine and BZE. In addition, in a relevant proportion cocaine alone—without BZE—was detected. Only in very few cases the inactive metabolite BZE—without cocaine—(1.6%) was confirmed in OF.

While specificity and sensibility of OF devices has been analyzed in various studies [7,9,10,22,23], there is always concern for police and policymakers when false positives occur. Importantly, according to Spanish regulations, drivers are fined when cocaine (and not BZE) is detected in OF. We have not tried to compare device performance: the occurrence of cases with confirmed positive results for BZE without cocaine (false positive cases), was 0.65%, 0.90% and 1.04% for the three devices used, but 1.76% when the device used was unknown. Because in nearly 9 out of 10 cases in which BZE (without cocaine) was detected, another illicit drug was confirmed (Table 1), in practice and when looking to the fact of whether the driver should be fined or not, in very few cases (55 cases out of 31,707) traffic police are facing the fact of OF confirmation on BZE (without any other substance).

There is limited information about cocaine and/or BZE concentration in OF from drivers who tested positive for these substances. OF concentrations of cocaine and BZE were higher when both substances were detected together than when cocaine—without BZE—and BZE—without cocaine—were detected. An important dispersion in cocaine and BZE concentrations was observed.

The pharmacokinetics of cocaine has been studied in various ways [12,24,25,26,27,28,29,30]. In controlled administration studies, cocaine was identified in OF after smoking, intravenous, intranasal and oral administration [8,24,25]. Cocaine is a weak base and is subject to OF ion trapping [6,8]. From the other side, the use of cocaine leads to reduced salivary volume (dry mouth) [8]. Furthermore, as reported [8], smoked crack cocaine, insufflation of cocaine hydrochloride, and oral cocaine use can lead to a contamination of the oral cavity. This way has reported high OF levels compared to concentrations that occur in blood [6,8,31]. Therefore, the correlation between OF and blood cocaine concentrations is a matter of concern [28,29,30,31,32,33,34], and ratios close to 20 have been described [8,32,33].

The limitations of the data analysis presented here have previously been described in detail [5,13,14]. Data on alcohol were not available. In the current practice of the Spanish traffic police, when an alcohol breath test is positive, screening for drugs is not performed (although this is not always the case). Consequently, our results do not allow for the assessment of the important issue of concomitant use of cocaine and alcohol. Information on gender and age was only regularly available for the year 2016. Finally, concerns about the quality of the evidence may arise, as this study was conceptually observational in nature. Additionally, differences between countries regarding the frequency of driving with the presence of cocaine could exist. There is also a lack of information on drivers with negative roadside drug tests, which precluded comparison between positive and negative cases. This study was representative of those positive for roadside drug testing but not representative of the general population of drivers in Spain.

## 5. Conclusions

This study provides real-world evidence on driving with the presence of cocaine (and/or BZE) and other driving-impairing substances by drivers in Spain: Positivity for cocaine represented an important proportion among Spanish drivers who tested positive for drugs. Of note, polysubstance use was observed in over 8 out of 10 positive cases for cocaine and/or BZE. It is well known that there is a high risk for fatal and serious injuries from road accidents when driving with the presence of various drugs [2,3,4]. The implementation of roadside drug testing in association with an efficient punitive system could be an efficacious public health intervention for maintaining safe driving [1,5,13]. Drivers who use drugs must perceive that the risk of detection is high, and this is particularly important if polysubstance use is taken into account [5,13,35].

## Figures and Tables

**Table 1:** Drivers with confirmed positive oral fluid tests for cocaine and/or benzoylecgonine (years 2011 to 2016).

	Cocaine and Benzoylecgonine	Cocaine without Benzoylecgonine	Benzoylecgonine without Cocaine
Number of positive roadside drug tests to any drug carried out 2011 to 2016	65,244	65,244	65,244
Drivers with a positive test for…. *n* (%)	25,773 (39.50)	5436 (8.33)	498 (0.76)
Cocaine and/or Benzoylecgonine Alone *n* (%)	4707 (18.26)	422 (7.76)	55 (11.05)
In combination with other drugs	21,066 (81.74)	5014 (92.24)	443 (88.95)
Tetrahydrocannabinol	17,572 (68.19)	4715 (86.74)	392 (78.71)
Opioids	5355 (20.78)	1220 (22.44)	78 (15.66)
Amphetamine-like substances	4320 (16.76)	233 (4.29)	59 (11.85)
Benzodiazepines	1843 (7.15)	166 (3.05)	27 (5.42)
Zoplicone, zolpidem	51 (0.20)	5 (0.09)	0 (0)

**Table 2:** Concentration of cocaine and/or benzoylecgonine in the oral fluid of drivers with a confirmed positive test (years 2011–2016).

	Cocaine and Benzoylecgonine		Cocaine without Benzoylecgonine	Benzoylecgonine without Cocaine
	Cocaine	Benzoylecgonine		
Mean (SD)	780.60 (4364.83)	338.72 (2540.41)	16.57 (20.55)	38.55 (94.21)
Median (Q1–Q3)	249.30 (55.30–405.00)	137.90 (27.80–405.00)	11.00 (7.23–19.29) *	9.90 (6.80–24.25) **

* Kruskal-Wallis H test: 6464.776, *p* < 0.0001; ** Kruskal-Wallis H test: 387.278, *p* < 0.0001. Abbreviations: SD, standard deviation; Q quartile.

## Data Availability

Restrictions apply to the availability of these data. Data was obtained from Spanish traffic authorities (Dirección General de Tráfico—DGT) and are may request from at unidad.investigacion@dgt.es (DGT).

## Supplementary Materials

The following are available online at https://www.mdpi.com/article/10.3390/ijerph18105371/s1, Figure S1: Distribution of medians and interquartile ranges for oral fluid cocaine concentrations, by age, for cocaine-positive cases. Figure S2: Distribution of medians and interquartile ranges for oral fluid benzoylecgonine concentrations, by age, for benzoylecgonine-positive cases. Table S1: Oral fluid drug testing devices used between 2011 and 2016, substances detected and cutoffs. Table S2: Roadside drug tests performed between 2011 and 2016 and the gender distribution of the Spanish population and the Spanish driving population. Table S3: Cocaine and/benzoylecgonine concentration deciles for positive tests between 2011 and 2016.
