# Supplementary material for: Positivity to Cocaine and/or Benzoylecgonine in Confirmation Analyses for On-Road Tests in Spain
Source: Int J Environ Res Public Health. 2021 May 18;18(10):5371. doi: 10.3390/ijerph18105371 (PMC8157855; doi:10.3390/ijerph18105371)
Supplement: Supplementary file 1 [file ijerph-18-05371-s001.zip › ijerph-1190369-supplementary.pdf]

Table S1. Oral fluid drug testing devices used between 2011 and 2016: substances detected and cut-offs [14].

**Oral fluid drug device**	**Analyte group**[a] **(Confirmed analyte)** Cut-off (ng/mL)					**Year range**	**Tests performed** (n)‡
	**Cannabis** (delta-9-tetrahydrocannabinol)	**Cocaine** (Cocaine*, Benzoylecgonine**)	**Amphetamine** (d-amphetamine)	**Methanphetamine** (Methamphetamine, MDMA***)	**Opioids** (Morphine, Codeine****)		
Dräger DrugTest® 5000	25.0	20.0*	50.0	35.0<br>75.0***	20.0	2011-2016	44012
Alere™ DDS®2 Mobile Test System	25.0	30.0**	50.0	50.0	40.0	2015-2016	13055
DrugWipe® 5S	30.0	30.0**	50.0	25.0<br>25***	10.0****	2015-2016	6531

[a] Data provided by Spanish National Traffic Agency: points of sensitivity of the investigative teams. Meeting GT 47. Madrid

MDMA= 3,4-metilendioxi-metanphetamine

‡Between 2011 and 2016, the roadside drug-testing device was unknown for 1646 tests.

Table S2. Roadside drug tests performed between 2011 and 2016 and gender distribution of the Spanish population and the Spanish driving population [14].

**Year (n)**	**Test performed (Confirmed tests at laboratory)**	**Gender distribution of test confirmed at laboratory**			**Age distribution of test confirmed at laboratory**		**General population***		**Driver population****		**Cocaine and/or Benzoylecgonine positive results**
	**N**<br>**(n)**	**Male**<br>n<br>(%)	**Female**<br>n<br>(%)	**Unknown**<br>n<br>(%)	**Known**<br>n<br>(%)	**Unknown**<br>n<br>(%)	**Total**<br>n	**Male**<br>**Female**<br>n<br>n	**Total**<br>n	**Male**<br>**Female**<br>n	**Total**<br>n<br>(%)
**2011 to 2016**	**179,645**<br>(65,244)	34,691<br>(53.2)	1,338<br>(2.1)	29,215<br>(44.8)	35,073<br>(53.8)	30,171<br>(46.2)					31,707<br>(48.60)
**2011**	**743**<br>(62)	35<br>(56.5)	2<br>(3.2)	25<br>(40.3)	0	62<br>(100)	46,818,216	23,104,303<br>23,711,613	26,133,510	15,545,721<br>10,587,789	24<br>(38.71)
**2012**	**3,487**<br>(**1**,087)	720<br>(66.2)	24<br>(2.2)	343<br>(31.6)	0	1,087<br>(100)	46,727,90	23,017,758<br>23,710,132	26,323,971	15,606,700<br>10,717,271	557<br>(51.24)
**2013**	**4,563**<br>(2,017)	22<br>(1.1)	1<br>(0.05)	1,994<br>(98.9)	0	2,017<br>(100)	46,512,199	22,877,461<br>23,634,738	26,401,660	15,616,630<br>10,785,030	1,001<br>(49.63)
**2014**	**29,643**<br>(9,991)	19<br>(0.2)	3<br>(0.03)	9,969<br>(99.8)	0	9,991<br>(100)	46,449,565	22,826,546<br>23,623,019	26,217,202	15,317,999<br>10,869,203	4,315<br>(4.19)
**2015**	**76,040**<br>(25,966)	8,969<br>(34.5)	316<br>(1.2)	16,681<br>(64.2)	8,952<br>(34.5)	17,014<br>(65.5)	46,440,099	22,807,464<br>23,632,635	26,350,036	15,362,190<br>10,987,846	12,994<br>(50.04)
**2016**	**65,169**<br>(26,121)	24,926<br>(95.4)	992<br>(3.8)	203<br>(0.8)	26,121<br>(100)	0	46,527,039	22,834,227<br>23,692,812	26,514,026	15,377,544<br>11,136,482	12,816<br>(49.06)

Table S3. Cocaine and benzoylecgonine concentration deciles for positive tests between 2011 and 2016.

Deciles	Cocaine			Benzoylecgonine		
	**Cocaine** (all cases)	**Cocaine and benzoylecgonine positive cases**	**Cocaine** (without BE)	**Benzoylecgonine**	**Cocaine and benzoylecgonine positive cases**	**BE** (without cocaine)
		**Cocaine**			**BE**	
	**(ng/mL)**	**(ng/mL)**	**(ng/mL)**	**(ng/mL)**	**(ng/mL)**	**(ng/mL)**
**10**	9,60	20,40	5,80	8,88	9,26	5,30
**20**	18,30	41,00	6,70	17,80	19,20	6,18
**30**	33,80	74,30	7,90	36,88	39,60	7,20
**40**	64,80	135,80	9,20	71,10	75,80	8,20
**50**	131,92	249,30	11,00	132,00	137,90	9,90
**60**	276,10	405,00	13,45	228,31	236,90	13,24
**70**	405,00	405,00	17,00	385,34	394,39	18,91
**80**	405,00	405,00	22,10	405,00	405,00	32,52
**90**	405,00	405,00	32,30	405,00	405,00	65,97

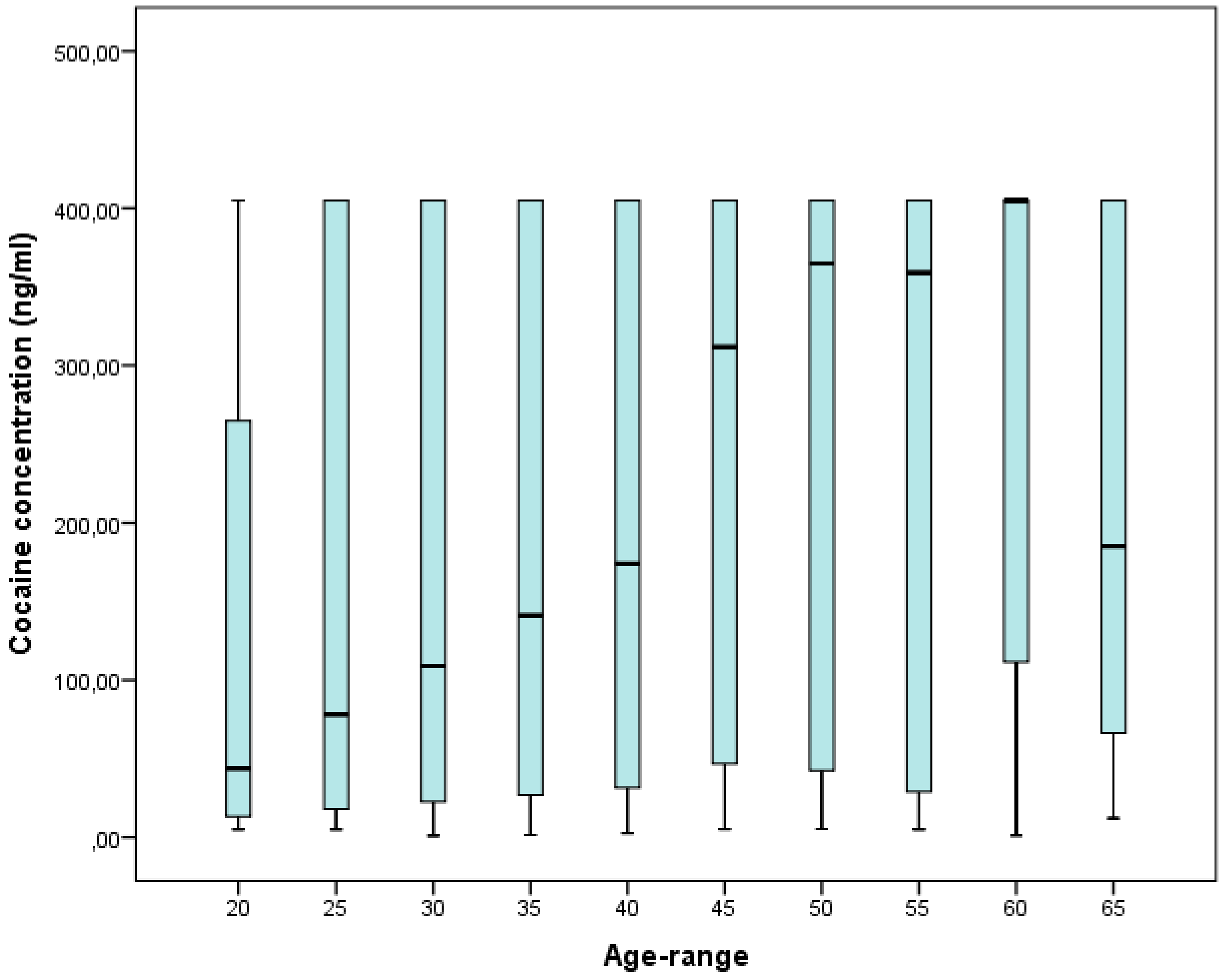

X-axis=5-year age distribution. Y-axis=cocaine concentration (ng/mL).

Figure S1. Distribution of medians and interquartile ranges for oral fluid cocaine concentrations, by age, for cocaine-positive cases.

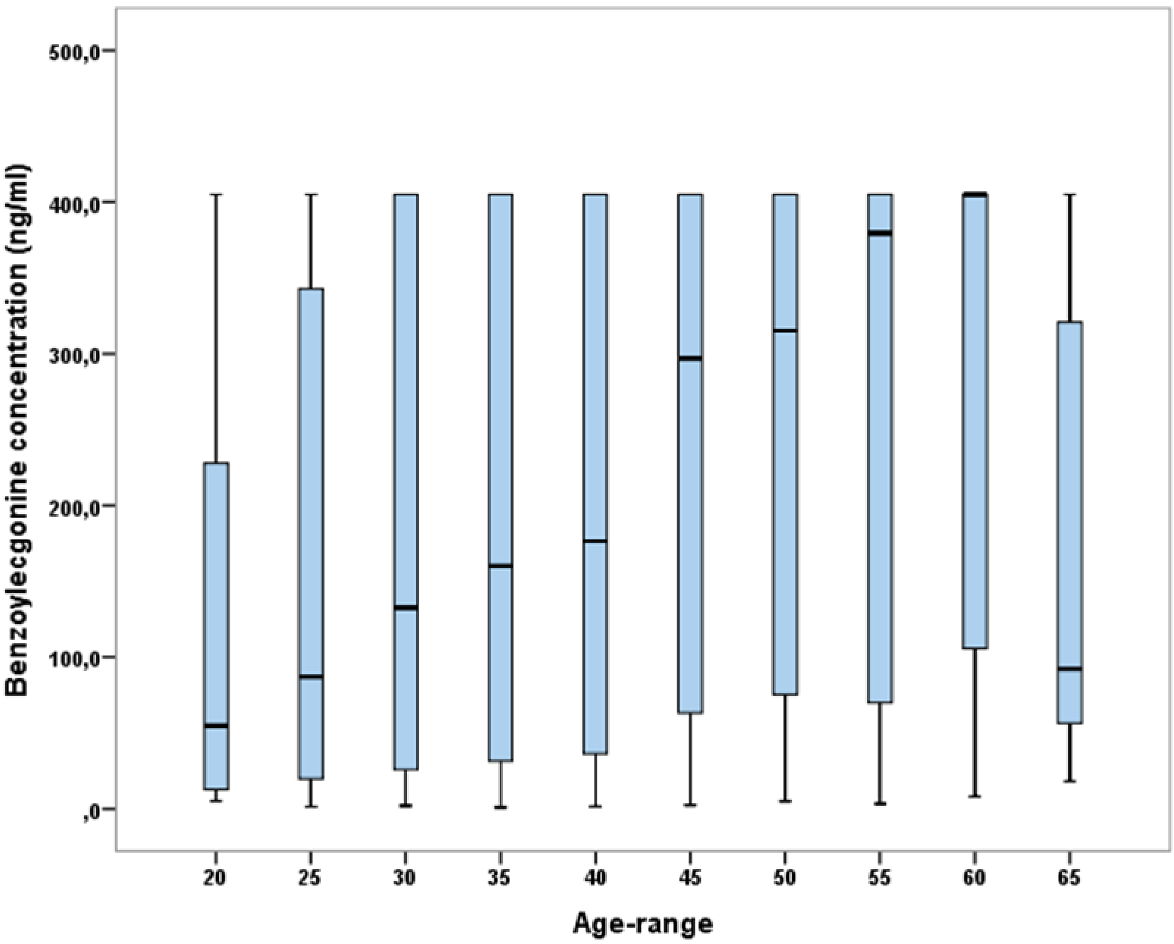

X-axis=5-year age distribution. Y-axis=benzoylecgonine concentration (ng/mL)

Figure S2. Distribution of medians and interquartile ranges for oral fluid benzoylecgonine concentrations, by age, for benzoylecgonine-positive cases
